# U.S. Science Takes a Shot in the Pocketbook

**DOI:** 10.1100/tsw.2001.44

**Published:** 2001-04-13

## Abstract

Nature leads this week with a story about the Smithsonian's decision to close two research centErs. Science kicks off with an analysis of the Bush administration's recently released science budget request.

The U.S. Smithsonian Institution, often called the "nation's attic," is best known for its vast collection of historical odds-and-ends. But it also supports research in everything from astronomy to zoology. At least it used to. Last week, the institution announced plans to close two research centers employing approximately 350 people, reports *Nature* in its lead story. *Science* also covered this story in their number two slot. Slated for death are the Smithsonian Center for Materials Research and Education, which develops methods for conserving museum artifacts, and the Conservation and Research Center, which collaborates with the National Zoo on studies of endangered species. And many scientists believe that more closings are on the way, potentially pushing Smithsonian research on to the list of endangered species. The announcement is "the first shoe of probably many...this is a centipede," said an unnamed former Smithsonian researcher (presumably a biologist) in *Nature*. Disgruntled employees pinned blame for the closings on Larry Small, a former banker who was appointed last year as the first non-academic to serve as the Smithsonian's secretary, reported *Nature*. The overall Smithsonian budget, however, is expected to rise slightly, say *Nature* and *Science*, and the savings from the center closings will be used to fund additional "public services".

*Science* leads with a story about the Bush administration's proposed science budget request for 2002. *Nature* also covered this story in the number nine position. Although the new budget appears to be a boon to research, report both journals, for many disciplines it is a bust. On the boon side, the National Institutes of Health (NIH) will receive a whopping 13.5% increase, worth almost $3 billion, if the U.S. Congress adopts the Bush proposal. But if the proposal does pass Congress as is, 2002 will be a major bust for the U.S. Geological Survey (USGS), who can expect to see their budget fall by 8%, reports *Science*. Given Bush's emerging anti-environmental stance, it is perhaps not surprising that the hardest hit USGS divisions will be agency's Toxic Substances Hydrology and National Water-Quality Assessment (NAWQA) programs. Keeping with that theme, the National Oceanic and Atmospheric Administration — an important player in climate change and ocean research — will lose 10% of the $365 million that its main science office has to spend this year, report *Nature* and *Science*. The National Science Foundation (NSF), the Department of Energy's (DOE's) Office of Science, and NASA will all maintain their current levels of funding, but in this age of ballooning science costs a flat budget is really a budget cut, say scientists interviewed by *Nature* and *Science*. *Nature* also points out that Bush has asked for a $2.5 billion increase in military research spending, reversing a steady decline in military spending during the Clinton administration. Both journals note that Congress will almost certainly amend the budget, but whether they will boost funding remains to be seen.
